# Immune Alterations with Aging: Mechanisms and Intervention Strategies

**DOI:** 10.3390/nu16223830

**Published:** 2024-11-08

**Authors:** Weiru Yu, Yifei Yu, Siyuan Sun, Chenxu Lu, Jianan Zhai, Yumei Lei, Feirong Bai, Ran Wang, Juan Chen

**Affiliations:** Key Laboratory of Precision Nutrition and Food Quality, Department of Nutrition and Health, China Agricultural University, Beijing 100083, China; yuweiru0@163.com (W.Y.);

**Keywords:** aging, immune system, immunosenescence, immunotherapy, diet intervention

## Abstract

Aging is the result of a complex interplay of physical, environmental, and social factors, leading to an increased prevalence of chronic age-related diseases that burden health and social care systems. As the global population ages, it is crucial to understand the aged immune system, which undergoes declines in both innate and adaptive immunity. This immune decline exacerbates the aging process, creating a feedback loop that accelerates the onset of diseases, including infectious diseases, autoimmune disorders, and cancer. Intervention strategies, including dietary adjustments, pharmacological treatments, and immunomodulatory therapies, represent promising approaches to counteract immunosenescence. These interventions aim to enhance immune function by improving the activity and interactions of aging-affected immune cells, or by modulating inflammatory responses through the suppression of excessive cytokine secretion and inflammatory pathway activation. Such strategies have the potential to restore immune homeostasis and mitigate age-related inflammation, thus reducing the risk of chronic diseases linked to aging. In summary, this review provides insights into the effects and underlying mechanisms of immunosenescence, as well as its potential interventions, with particular emphasis on the relationship between aging, immunity, and nutritional factors.

## 1. Introduction

The global trend of population aging has heightened the focus on age-related diseases [1], spurring investigations into delaying or reversing the detrimental effects of aging. Aging is a ubiquitous and complex physiological process driven by the progression of time and environmental interactions, resulting in structural and functional alterations at the molecular, cellular, and systemic levels throughout all biological systems [2]. The immune system, as a critical defense barrier, identifies antigens and mounts responses crucial for maintaining health. The intricate impact of aging on the immune system impairs physiological function and disrupts homeostatic equilibrium, markedly elevating the risk of degenerative diseases and mortality [3]. Immune cells are generally considered to act as monitors in the aging process and are primarily responsible for the removal of harmful senescent cells. The progressive decline in various immune functions during aging is termed “immunosenescence” [4]. This age-related deterioration of the immune system heightens vulnerability to diseases as a consequence of immunosenescence. The aging immune system can be targeted through various interventions, including lifestyle interventions, pharmacological interventions, and immunotherapies [5]. Among these, nutritional intervention stands out as the safe and promising approach for personalized treatment. In short, a thorough understanding of the interactions between aging and immune function is essential for developing targeted interventions. This review encapsulates the age-associated immune function changes, their health implications, the underlying biological mechanisms, and the potential strategies for intervention.

## 2. Immunological Changes During Aging

The replenishment of mature immune cells throughout an organism’s life involves processes centered in the bone marrow and thymus, which are crucial for sustaining immune cell development and function. However, the functionality of these organs declines with age, which subsequently reduces their capacity to renew the pool of immune cells. The concept of ‘immunosenescence’ was introduced for the first time, and research into immunosenescence has since been initiated [6].

### 2.1. The Composition of the Immune System

The immune system is divided into the innate and adaptive components. Innate cells include phagocytes and natural killer (NK) cells, while adaptive cells consist of T and B cells, all originating from hematopoietic stem cells. Upon pathogen entry into the bloodstream, the complement system activates, tagging invaders for destruction. Phagocytes use pattern recognition receptors (PRRs) to detect and engulf pathogens. If innate defenses fail, adaptive immunity activates. Antigen-presenting cells (APCs) migrate to lymph nodes, presenting antigens to T cells, which recognize them through T cell receptors (TCRs). This triggers helper and cytotoxic T cells, as well as B cells that produce neutralizing antibodies. Memory T and B cells ensure a faster and stronger response upon re-exposure to the same antigens [7].

### 2.2. Impact of Aging on Innate Immune System

#### 2.2.1. Neutrophils

Neutrophils are crucial immune cells that combat infections through phagocytosis, secretion of antimicrobials, and neutrophil extracellular traps (NETs) [8,9]. Some studies indicate that while neutrophil development and proliferation remain stable with age [10], their functional efficiency declines, as evidenced by reduced phagocytic capability, chemotaxis, cytokine production, and oxidative burst [11]. This reduction extends to decreased phagocytic and bactericidal activities, as observed in various aging models [12,13]. Moreover, a recent multi-omics analysis revealed significant transcriptomic and epigenomic alterations in neutrophils due to aging, implicating the aged bone marrow microenvironment in the dysregulation of these cells [14]. In vivo studies in mice have also established a direct correlation between neutrophil senescence and increased pro-inflammatory activity [15]. Such alterations in neutrophils heighten the susceptibility to infections and inflammatory diseases in the elderly [16,17]. Moreover, some research has demonstrated that aging interferes with transepithelial migration of neutrophils in injured tissues via a process regulated by CXC-chemokine ligand 1 (CXCL1), leading to improper neutrophil trafficking and consequent remote organ damage [18].

#### 2.2.2. Macrophages

Macrophages, which are key phagocytic cells, rapidly migrate to infection or injury sites, where they recognize, engulf, and digest pathogens and cellular debris. They eliminate these pathogens through phagocytosis and the release of antimicrobial substances [19]. Aging mice showed increased CD38 in liver and adipose tissue, which was mainly derived from pro-inflammatory M1 macrophages, indicating that the differentiation balance of macrophages was destroyed [20]. Aging-related changes in tissue integrity, such as increased extracellular matrix stiffness [21] and structural loss [22], may alter macrophage niches and their effector signals. In aging mice, diminished DNA repair and increased senescence in macrophages promote inflammation and in turn accelerate aging [23]. Caloric restriction reverses the pro-inflammatory polarization of aged M1 macrophages, enhances anti-inflammatory phenotypes, and modifies macrophage–stromal interactions [24]. Furthermore, by restarting glucose metabolism in macrophages, inflammation can be reduced, immune function can be restored, and aging can be reversed. As a result, the cognitive performance in older individuals can be improved [25].

#### 2.2.3. Dendritic Cells (DCs)

Dendritic cells (DCs) are key antigen-presenting cells that initiate and regulate immune responses. With high migratory capacity, immature DCs rapidly move to infection or injury sites, where they capture pathogens and present them to T cells, activating adaptive immunity [26]. Research indicates that aging differentially impacts dendritic cell subtypes in mice; conventional type 1 DCs diminish in the spleen with age, while type 2 DCs do not [27]. Aging impairs DCs’ phagocytic functions, notably reducing their ability to perform efferocytosis of apoptotic cells [28]. Additionally, aging impairs DCs’ migration and cytokine production (IL-1β and CCR7). The decline in the number, along with the deterioration of their phagocytic and migratory functions, leads to a reduced capacity for activating downstream immune cells, thereby impairing their antigen presentation capability. In response, a vaccine adjuvant, DC hyper-activators, was developed to restore DCs’ migration, enhance anti-tumor CD4^+^ T cell responses, and bolster tumor immunity in aged mice [29].

#### 2.2.4. Natural Killer (NK) Cells

Natural killer (NK) cells are large granular lymphocytes that are distinct from T and B cells and possess both cytotoxic and immunomodulatory functions. They play a crucial role in controlling malignant tumors and infections. While aging typically reduces the number of lymphocytes, NK cells are an exception, showing an increase in number but a decline in function. An analysis of age-related immune cell data revealed a significant decrease in total lymphocyte count after the age of 60, whereas the NK and NKT cell numbers increased significantly [30]. This may be due to the fact that chemokines, such as CCL2 and CCL4, which rise during aging, can recruit macrophages and NK cells [31]. Despite this numerical increase, aging diminishes NK cell cytotoxicity and cytokine production [32]. There is a shift in NK cell subpopulations, with a decrease in the proportion of CD56^bright^ cells and an increase in CD56^dim^ cells. The increased CD56^dim^ NK cells exhibit reduced proliferative capacity and impaired pathogen clearance, leading to diminished immunity [33]. Additionally, aged NK cells inadequately remove senescent fibroblasts, which release pro-inflammatory factors known as the senescence-associated secretory phenotype (SASP). These factors contribute to tissue degradation and propagate senescence, thereby accelerating skin aging [34].

### 2.3. Impact of Aging on Adaptive Immune System

#### 2.3.1. T Cells

T cells, produced in the thymus, include helper (CD4^+^) and cytotoxic (CD8^+^) subtypes. CD4^+^ T cells regulate immune responses by secreting cytokines, while CD8^+^ T cells directly eliminate infected or cancerous cells. Aging is associated with a reduction in naive CD8^+^ T cells, an expansion in memory subsets, and an increase in clonal expansions, reducing available TCR diversity [35,36]. As with CD8^+^ T cells, the proportions of naive CD4^+^ T cells decline, and the memory population expands with age in mice and humans [37,38]. This switch to a memory phenotype seems to be attributable to diminished thymic output and the cumulative effects of lifelong antigen exposure [35]. An increase in regulatory T cells (Tregs) in aging spleens and lymph nodes has also been observed [39,40]. A predictive model of immune aging in a Chinese cohort showed decreases in naive T cells, increases in central and effector T cells, and stability in programmed death protein (PD)-1-associated subpopulations [41]. In addition, it has been reported that CD4^+^ T cells can stop the increase in senescent cells once a person is aging. The higher the number of CD4^+^ T cells in a tissue sample, the lower the number of aging cells in aging skin [42]. Aging also leads to increased membrane rigidity, weakening the formation of the immune synapse and disrupting the signaling pathways essential for T cell activation, such as TCR and CD28. Additionally, aging causes the loss or reduced expression of CD28, altering T cell fate, reducing IL-2 production, and impairing the immunometabolic shift from oxidative phosphorylation (OXPHOS) to aerobic glycolysis. These metabolic changes, driven by altered mTOR signaling, result in insufficient energy production [43]. Overall, aging is associated with a decline in naive T cells and profound differentiation into memory phenotypes as co-stimulatory molecules (such as CD28 and CD27) are lost, leading to T cell senescence or exhaustion and impaired cytokine secretion. Aging T cells also exhibit characteristics such as mitochondrial dysfunction and epigenetic changes [44]. Additionally, CD57 and CD95 have been employed to identify senescent human T cells [45], and CD153 is commonly used to identify a minor population of senescent T cells in mice [46].

#### 2.3.2. B Cells

B cells play crucial roles in synthesizing antibodies, presenting antigens, and secreting cytokines, all of which are essential for immune defense. Their development and function are critical to the body’s capacity to respond to pathogens. However, aging significantly diminishes B cell production and functionality, contributing to decreased immunity in the elderly. Adaptive immune remodeling with age includes changes in B cell composition and functionality. Specifically, a unique subset termed ‘age-associated B cells’ (ABC) expands in the spleen and bone marrow of aged mice [47,48]. These cells, which are distinct from traditional naive and memory B cells, were identified as CD43^−^ CD21/CD35^−^ CD23^−^ B cells by Hao et al. [47] and as CD11b^+^CD11c^+^ B cells by Rubtsov et al. [48]. Notably, they express Tbx21, linking them to autoimmunity in a similar manner to that observed in lupus-like autoimmunity in mice [49,50]. Aging is associated with a more mature B cell state and a reduction in naive B cells, indicating a compromised cellular immune response to new antigens [51]. Furthermore, aging disrupts DNA interactions within the topologically associating domain (TAD) of the immunoglobulin heavy chain locus (IgH locus), reducing the diversity of the primary BCR repertoire. These alterations impair B cell development and function, contributing significantly to immune senescence [52,53].

### 2.4. Senescence-Associated Secretory Phenotype (SASP)

Cells may enter a senescent state as a consequence of various stressors, which further drives organismal aging. Cell cycle arrest is a typical feature of cellular aging; it leads to a stable, terminal proliferative halt and the development of a SASP [54]. The SASP defines the secretion of various cytokines, chemokines, growth factors, proteases, and lipids by senescent cells. This concept was first introduced by Krtolica et al. in 2001 [55]; they proposed that senescent cells secrete factors into their microenvironment, thereby potentially modulating biological activities both locally and systemically. In 2008, the SASP, also known as the senescence-messaging secretome, was independently characterized by various laboratories as primarily consisting of pro-inflammatory and growth-stimulating proteins [56,57]. This secretion leads to chronic inflammation and tissue damage in organisms. The composition of the secretome varies depending on the trigger of senescence, and the proportion of senescent cells in very old primates is estimated to range from 5 to 20% [58], contributing to age-related diseases. The SASP can also interact with immune cells, creating feedback loops that exacerbate tissue damage [44].

**Figure 1:**
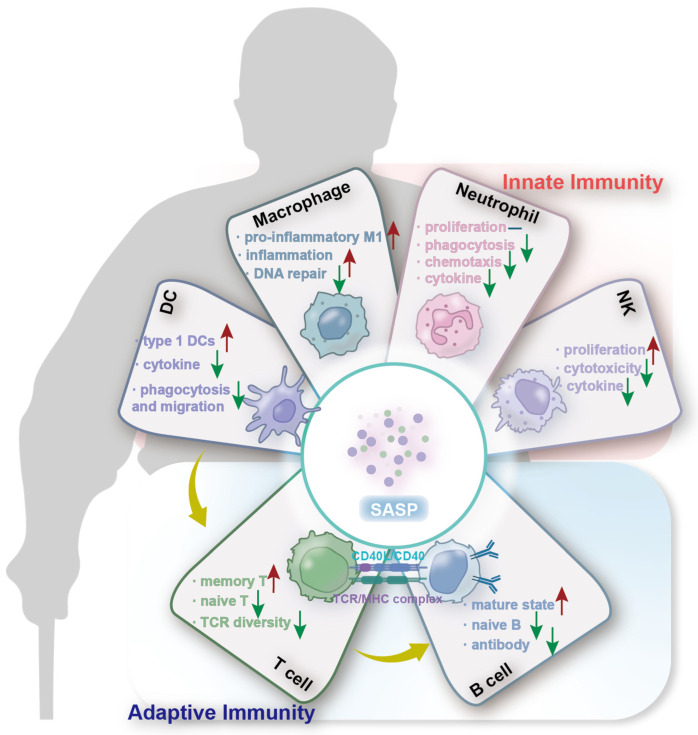
The effects of aging on different immune cells in the intrinsic and adaptive immune systems. Changes in the immune system involve the decline of both innate and adaptive immunity, along with the development of the senescence-associated secretory phenotype (SASP). Aging leads to alterations in the number and function of immune cells, with the adaptive immune system being more significantly affected. For innate immunity, the percentages of pro-inflammatory macrophages (M1) and type 1 DCs increase during aging; the phagocytosis and cytokine secretion decrease in both DCs and neutrophils; the cytotoxicity of NKs also decline in old individuals. For adaptive immunity, both T cell and B cell populations decline in the elderly, with a concomitant reduction in TCR diversity and impaired antibody production. The red upward arrow indicates an increase, the green downward arrow indicates a decrease, and “—” represents no change.

In summary, there are fewer immune cells, weaker immune responses, less antibody production, and an increase in the immune aging marker in various immune cells with aging [59] (Figure 1).

## 3. Mechanism of Aging Affecting Immunity

### 3.1. Mechanism at the Molecular Level

#### 3.1.1. Thymic Involution

One of the most well-documented changes in the aging immune system is the involution of the thymus, which is crucial for developing a diverse and selectively refined T cell repertoire [60,61]. This process results in a reduced generation of naive T cells reaching the periphery, a compensatory expansion of memory T cells, and decreased diversity in the peripheral T cell repertoire, impairing pathogen detection. This aligns with observed trends in T cell aging. In murine thymic tissues, numerous nucleic acid-binding proteins (NABPs), which play key roles in immune regulation, immune cell activity maintenance, and cancer control, exhibit dynamic changes with age [62]. However, in murine spleen tissues, the binding activities of NABPs related to immune and defense functions remain relatively stable, possibly due to the spleen’s inherent regenerative capacity and consistent phenotypic maintenance. Reversal of thymic atrophy has been demonstrated using intra-thymic injections of thymic epithelial cells (TECs), a technique that enhances autoreactive thymocytes, reducing inflammation and T cell aging [63].

#### 3.1.2. Inflammaging and Oxidative Stress

Originally perceived as a biomarker for aging, inflammaging is now recognized for its association with increased risks of age-related multimorbidity and mortality [64,65]. This condition results from a combination of age-related deficits, including enhanced gut permeability, chronic infections, and an accumulation of senescent cells, which collectively drive the overproduction of the pro-inflammatory cytokines characteristic of the SASP [66]. Elevated reactive oxygen species (ROS) levels induce HIF1-α expression, leading to enhanced glycolysis and TNF-α secretion [67]. For a rather small cohort of people, an increase in mitochondrial ROS production by CD8^+^ T lymphocytes has been observed [68]. Additionally, in vitro studies have demonstrated that telomere shortening within this CD8+ subset can be mitigated by treatment with a ROS scavenger, underscoring a potential causal relationship between ROS accumulation and T cell aging [68]. In murine models, peritoneal leukocytes, particularly macrophages, exhibit an age-dependent increase in ROS levels, further implicating oxidative stress in the aging process [69].

#### 3.1.3. DNA Damage

During aging, nuclear DNA in humans and model organisms accumulates somatic mutations, gene copy number variations, and chromosomal aneuploidies. These changes can affect functionally essential genes, leading to altered cells, tissue abnormalities, and organismal deficiencies, which contribute to aging and age-related pathologies [70,71]. In hematopoietic cells, this accumulation results in spontaneous DNA damage in immune cells and an increased expression of senescence and SASP markers in various cell types, including B cells, NK cells, CD4^+^ and CD8^+^ T cells, and monocytes/macrophages. This pattern resembles that observed in 2-year-old naturally aged mice [72,73]. These findings suggest that DNA damage may lead to increased cellular senescence in immune cells with age, potentially triggering secondary senescence through SASP factor secretion. Although all species have evolved complex DNA repair mechanisms to mitigate unavoidable genetic damage and maintain cellular homeostasis, these mechanisms become less effective with age. Consequently, the persistent accumulation of genomic damage increases susceptibility to cancer and other age-related diseases [74,75]. Moreover, deficiencies in DNA repair mechanisms have been implicated in aging and cancer. Disruptions in DNA repair processes can accelerate aging in experimental models [76].

#### 3.1.4. Telomere Shortening

Telomere shortening prevents replicative DNA polymerase from fully replicating telomeric regions. Without intervention, successive cell divisions exacerbate telomere erosion, leading to genomic instability and ultimately resulting in permanent cell cycle arrest (senescence) or cell death [77].

#### 3.1.5. Mitochondrial Function Changes

Mitochondria are crucial for bioenergetics, cellular metabolism (including NAD/NADH ratio maintenance), and various other functions. They act as signaling hubs, producing and responding to ROS and calcium spikes [78,79]. These signaling pathways become dysregulated in aged T cells [80]. As cells age, mitochondrial homeostasis is disrupted, leading to the release of mitochondrial DNA (mtDNA) into the cytoplasm. Age-related mutations in mtDNA impair mitochondrial oxidative phosphorylation (OXPHOS) functions [81]. In murine models, doxycycline-induced mutations result in decreased mtDNA levels, altered mitochondrial gene expression, and destabilization of OXPHOS complexes, contributing to skin aging and hair loss [82]. Studies on mice lacking DNA polymerase γ reveal that mtDNA mutations directly contribute to aging and related pathologies. These mice show accelerated aging and reduced lifespan, largely due to mtDNA deletions [83].

### 3.2. The Role of Molecular Signaling Pathway

Aging profoundly affects the immune system, increasing vulnerability to infections and chronic diseases. Key pathways, including mTOR, cGAS-STING, NF-κB, and JAK-STAT, regulate functions like cell growth, inflammation, and immune responses. Dysregulation of these pathways with age drives immune decline, emphasizing their role in immunosenescence.

#### 3.2.1. mTOR Signaling

The mammalian target of rapamycin (mTOR), a member of the PI3K-related kinase (PIKK) family, regulates cellular growth and metabolism in response to nutrients and hormones. It is a highly conserved pathway influencing aging across species [84]. Dysregulation of mTOR signaling, often due to nutrient insufficiency, is closely associated with human aging and related diseases [85]. Impaired mTOR signaling affects T cell activation, differentiation, and immune response, underscoring its central role in aging [86]. In addition, mTOR is a key regulator of aging in organisms ranging from yeast to mammals. The extension of lifespan through the pharmacological inhibition of mTORC1 with rapamycin or genetic modification in S6K1 mutant mice highlights its significance [87]. Recent human studies have further demonstrated that mTOR inhibition with RAD001 enhances antibody titers to influenza vaccination and reduces PD-1 expression on CD4^+^ and CD8^+^ T cells [88].

#### 3.2.2. cGAS-STING Signaling

Mitochondrial imbalances are significant in aging and neurodegenerative disorders, with mtDNA playing a central role in the activation of the cGAS-STING pathway. This pathway’s activation during aging is a critical driver of inflammation, aging, and functional decline. Elevated phosphorylation levels of STING, TBK1, and IRF3 in Alzheimer’s disease patients and aged mouse brains suggest the cGAS-STING pathway activation [89]. Administering H-151, a small molecule inhibitor of STING, to aging mice decreases the expression of age-associated immune genes (B2m, Il1b, Il6, Tnf and Ccl5) and inflammatory cell accumulation, enhancing muscle strength and cognitive functions. Moreover, similar results were obtained when the pathway was inhibited or the gene encoding the STING protein was knocked out [90].

#### 3.2.3. NF-κB Signaling

The NF-κB pathway, a fundamental regulator of innate immunity found across various species from insects to vertebrates, plays a crucial role in inflammaging. This signaling pathway orchestrates the intracellular responses to age-related DNA and cellular stress, implicating it in aging and associated diseases [91]. NF-κB, along with C/EBP and p53, governs the SASP, influencing a range of biological processes, such as immune responses, inflammation, stress reactions, B cell development, and lymphoid organogenesis [58]. Additionally, in the adaptive immune system, similar regulatory axes involving Sirt1-HIF-1α and Sirt1-NF-κB modulate metabolism and inflammation in aging T cells [92,93].

#### 3.2.4. JAK-STAT

**Figure 2:**
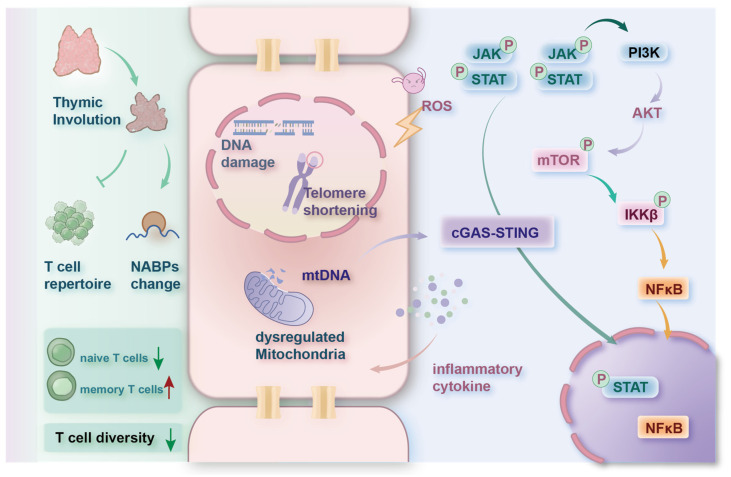
Multiple factors contribute to the aging of the immune system. These include thymic involution, inflammatory damage, DNA damage, telomere shortening, and mitochondrial dysfunction. The overall oxidation level increases with the higher level of inflammatory cytokines, activating pathways like NF-κB. The release of mtDNA from dysregulated mitochondria triggers the cGAS-STING pathway. The interaction of multiple pathways aggravates SASP. The red upward arrow indicates an increase, the green downward arrow indicates a decrease.

The JAK-STAT pathway is crucial for the differentiation and development of immune cells and is modulated by a variety of cytokines. It plays a key role in T cell differentiation; IFN and IL-12 promote Th1 cell differentiation through STAT1 and STAT4, while IL-4 activates Th2 cells via STAT3 upregulation of GATA6. Similarly, IL-6 and TGF-β trigger Th17 cell differentiation through STAT17-mediated activation of RORt, and both IL-6 and IL-12 influence T follicular helper cell differentiation via STAT3-driven Bcl-6 expression. IL-2 supports Treg cell differentiation by coordinating STAT5A/B with Foxp3 [94]. The JAK-STAT signaling pathway also mediates IL-6 to activate the MAPK enzyme and transcription factor NF-κB and to regulate inflammation and immune function [95]. In addition, the JAK inhibitor ruxolitinib has been shown to decrease the size of the secretome from senescent cells, underscoring the pathway’s relevance to aging [96] (Figure 2).

## 4. Aging Immune System and Aging-Related Diseases

**Figure 3:**
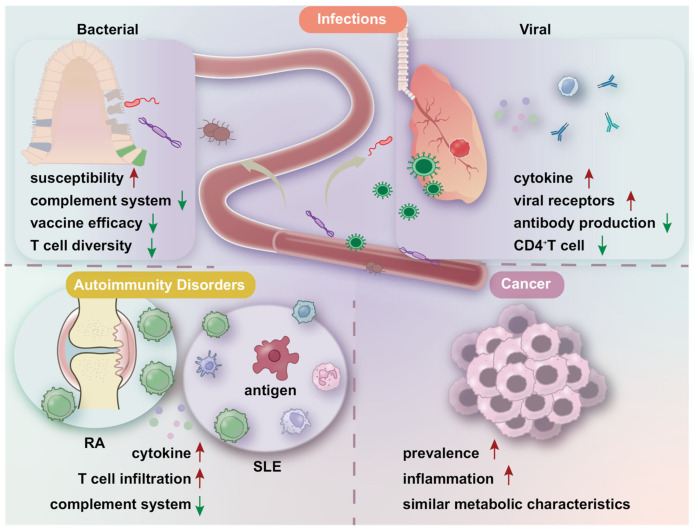
Aging affects the immune system and leads to age-related diseases. During aging, the likelihood of infection increases due to the deterioration of the immune system. This process is characterized by an enhanced inflammatory response, reduced T cell diversity, and diminished antibody production, which collectively increase vulnerability to bacterial and viral infections. Additionally, as aging progresses, the immune system’s tolerance to self-antigens decreases, and immune cell infiltration occurs, contributing to the development of autoimmune diseases. Furthermore, compromised immune functions during aging provide a conducive environment for cancer development. The red upward arrow indicates an increase, the green downward arrow indicates a decrease.

Age-related changes in immune system function can have profound effects on the physiology of elderly organisms, contributing to the onset of infections, autoimmune diseases, and cancer. The decline in immune function weakens the body’s defense against tumor cells and pathogens, while the chronic inflammatory state increases the risk of autoimmune diseases (Figure 3).

### 4.1. Increased Susceptibility to Infections

#### 4.1.1. Bacterial Infections

In addition to the previously noted decline in immune competence among older adults, the complement system, which is essential for rapid response to bacterial infections, is also compromised. Dysregulation of this system is frequently associated with and exacerbates age-related disease episodes [97]. Immune senescence increases the susceptibility of older adults to acute viral and bacterial infections, with mortality rates from these infections being threefold higher among elderly patients compared to younger adults [98]. During typical influenza seasons, approximately 90% of excess mortality occurs in individuals over the age of 65, with poor immune responses significantly reducing the efficacy of vaccines [99,100]. Throughout an individual’s lifespan, continuous division, differentiation, and exposure to foreign infectious agents may diminish the diversity of T cells, thereby reducing their effectiveness. In the aging population, transformations in regulatory T cells occur; naive-like regulatory T cells decrease, whereas memory-like regulatory T cells increase with age. These changes in regulatory T cells, which help control the T cell compartment, promote anti-inflammation [101].

#### 4.1.2. Viral Infections

Immunosenescence refers to the age-related alterations in both innate and adaptive immune systems, leading to diminished capacity to combat novel infections and fostering chronic inflammation [102]. This phenomenon also reactivates latent viruses such as the varicella-zoster virus, culminating in conditions like shingles and chronic neuralgia [103]. Infection with latent viruses such as cytomegalovirus (CMV) has also been shown to potentially play a role in immunosenescence by amplification of inflammation, and both CMV seropositivity and elevated CMV IgG antibodies are linked to phenotypic and functional changes in adaptive immunity, as well as an elevated risk of all-cause mortality [104]. These immunosenescent changes render older adults especially susceptible to SARS-CoV-2 and severe COVID-19 outcomes [105]. In the older individual cases of SARS-CoV, non-survivors exhibited heightened innate immune activation—specifically IFN-α and IFN-γ, CXCL10, CCL2, and downstream IFN-stimulated genes—yet showed deficient antibody production [105]. Early activation of the adaptive immune response by SARS-CoV-2 may disrupt the timely innate immune response necessary for rapid viral elimination, potentially leading to a detrimental cytokine storm [106]. In the immune defense against viruses, CD4 Th1 cells are crucial. During SARS-CoV-2 infections, the adaptive immune response is critical due to the virus’s ability to evade innate immunity [107]; however, CD4 cell counts are typically reduced in aged individuals. In addition, the Angiotensin Converting Enzyme 2 (ACE2) [108] and the adipokine enzyme DPP4 [109] enzymes are more abundant in older people than in younger people, and after viruses enter the cells by binding to them, aging in turn induces further upregulation of the receptors in the context of a reduced immune response, resulting in older people being more susceptible to viral attack.

### 4.2. Autoimmunity Disorders and Inflammatory Disorders

Aging is strongly associated with the development of autoimmune diseases. Immunosenescence diminishes the immune system’s pathogen defense while compromising self-tolerance, thereby increasing susceptibility to autoimmune reactions. Elderly individuals frequently exhibit chronic low-grade inflammation, or “inflammaging”, which accelerates the progression of age-related conditions, such as rheumatoid arthritis (RA) and systemic lupus erythematosus (SLE) [110]. Altered immune cell functions, particularly in T and B lymphocytes, further heighten the risk of autoimmunity. Impaired self-/non-self-recognition and immune tolerance loss contribute to the increased incidence of autoimmune disorders in aging populations.

#### 4.2.1. Rheumatoid Arthritis (RA)

Rheumatoid arthritis (RA) is a chronic, autoimmune inflammatory condition that can lead to disability and premature mortality. The age-specific prevalence of RA was highest among individuals aged 75–79, with 828.2 cases per 100,000 population in 2020 [111]. In RA patients, the levels of complement components C4d and C3bBbP were found to be 2–34 times higher than in the controls, aligning with data showing a correlation between complement C3 and C4 levels and age [112,113]. Longevity is negatively correlated with C3/C4 levels, suggesting that high C3 levels are detrimental to longevity. Furthermore, T cells in RA patients exhibit premature aging features, such as accumulation of CD28, telomere fragility, diminished DNA repair capacity, and excessive cytokine production [114]. These maladaptive T cells entering cellular senescence remain hyperactive and continuously secrete pro-inflammatory cytokines like TNF-α and IFN-γ, thereby perpetuating inflammation and accelerating aging due to deviation from homeostatic mechanisms.

#### 4.2.2. Systemic Lupus Erythematosus (SLE)

Systemic lupus erythematosus (SLE) is a complex systemic autoimmune disease characterized by high heterogeneity and intricate pathogenesis. Dysfunction in both innate and adaptive immune responses is evident in SLE patients and involves activation of inflammatory pathways by dendritic cell type I IFN secretion to drive disease progression [115], neutrophilic leukocyte iron death [116], disruption of the balance of macrophage differentiation subtypes [117], and aberrant T and B cell proliferation and differentiation [118,119]. It was found that older age significantly increases the risk of neuropsychiatric events in systemic lupus erythematosus, demonstrating the complex interplay between aging and SLE progression [120]. Additionally, aging disrupts the expression of complement system proteins and impairs the clearance of apoptotic material due to deficiencies in C1q, C4, and C2, further increasing the SLE risk [121]. The increase in brain age is associated with neuronal damage and cognitive impairment in SLE patients [122]. Recent research suggests that T cells infiltrating the choroid plexus could drive neuropsychiatric symptoms in lupus, which could link these immune cells to the age-related cognitive decline observed in SLE patients [123].

### 4.3. Cancer

Cancer prevalence increases with age, which is attributed to heightened cancer risk factors and the expansion of the aging population [124]. Li et al. report that 60% of all cancer diagnoses and 70% of cancer-related deaths occur in individuals aged 65 and older, with projections suggesting that by 2030, this age group will account for 70% of all cancer diagnoses [125]. Older adults diagnosed with cancer (>65 years) are more likely to exhibit comorbidities and age-related conditions compared to those without cancer [126]. Reciprocally, the onset of malignancy can exacerbate the decline in general health due to the systemic effects of cancer on distant organs, including the intestinal microbiota [127,128]. Moreover, cancer treatments, whether localized (surgery or radiotherapy) or systemic (chemotherapy, immunotherapy, etc.), induce organismal stress and inflammatory responses, accelerating the aging process [129,130]. Therefore, the interaction between aging and cancer is bidirectional. Specifically, research indicates that several aging characteristics, such as genomic instability, epigenetic alterations, chronic inflammation, and dysbiosis, are intimately associated with cancer determinants. However, other aspects of aging, like telomerase attrition and stem cell exhaustion, exhibit tumor-suppressing properties [131]. Additionally, aging and cancer share similar metabolic characteristics. In short, these positively or negatively regulated factors work together and may explain the peculiar association between cancer and aging with an increment of malignancies.

## 5. Interventions and Strategies to Improve Immune Function in Aging

Immune aging contributes to a vicious cycle that exacerbates the aging process itself, thereby increasing susceptibility to a broad spectrum of age-related diseases. Developing interventions that target the mechanisms of immune aging may offer new strategies for preventing these conditions. By understanding the pathways and factors contributing to immune deterioration, targeted therapies can potentially disrupt this cycle and enhance overall health in the elderly population.

### 5.1. Lifestyle Interventions

#### 5.1.1. Diet

**Figure 4:**
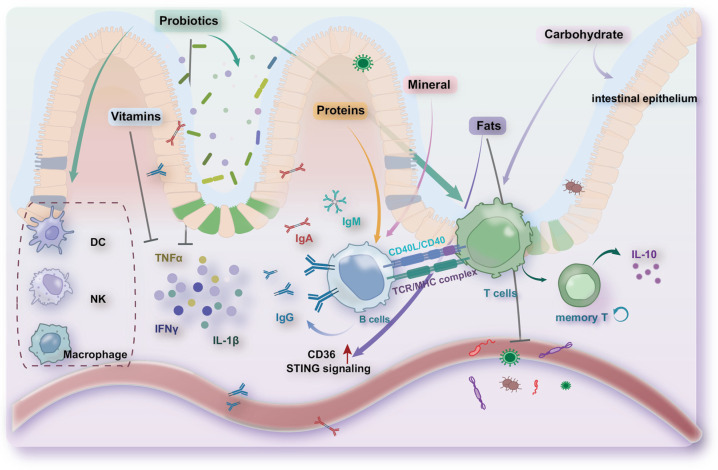
Dietary interventions, including proteins, carbohydrates, fats, vitamins, minerals, probiotics, and prebiotics, have significant effects on immune system function. Proteins enhance immunity by influencing T cell survival and differentiation, as well as by promoting B cell antibody production. Carbohydrates can strengthen innate immune responses and reduce cell apoptosis. The immunomodulatory effects of fats are primarily mediated by fatty acids, which enhance T cell antigen presentation, B cell proliferation, antibody production, and the antimicrobial activity of immune proteins. Vitamins regulate thymic and bone marrow homeostasis, enhancing the function of macrophages, dendritic cells, and T cells. Minerals play an essential role in modulating the production of immune regulators and antibodies. Probiotics and prebiotics modulate gut immunity by influencing the gut microbiota, reducing inflammation, and enhancing the function of various immune cells. The red upward arrow indicates an increase, the green downward arrow indicates a decrease.

With the advancement of research in the intersection of metabolism and immunology, there is increasing recognition of the critical role that nutrients play in modulating immune function. Dietary factors influence immune system functionality, thereby affecting the aging process and the onset and progression of diseases (Figure 4).

##### Macronutrients

Macronutrients, including proteins, carbohydrates, and fats, support metabolic activities and energy balance through distinct pathways.

Proteins and amino acids are fundamental to the immune system, playing significant roles in the development and function of immune tissues and organs. They are essential for the production of various immune cells and the synthesis of antibodies. Amino acids are transported into the cytoplasm via solute carrier (SLC) transporters, where they activate sensor proteins, such as mTOR, GCN2, and Sestrin. These pathways can directly activate the TCR-CD3 complex or undergo further metabolism to influence T cell development and survival. For instance, an increase in arginine can restrict T cell differentiation, helping maintain T cells in a memory state under homeostatic conditions. Moreover, supplementation with L-arginine has been shown to significantly enhance the survival of activated CD4^+^ and CD8^+^ T cells in vitro and in mice, thereby improving antitumor activity [132]. During the late differentiation phase of T cell activation, asparagine (Asn) restriction enhances the proliferative and effector capacities of CD8^+^ T cells by promoting metabolic fitness and antitumoral functionality through an NRF2-dependent stress response [133]. For B cells, threonine (Thr) further influences the differentiation and function of monocytes, while leucine (Leu) is transported into B cells via SLC7A5, targeting mTORC1 to promote B cell differentiation and support the production of IgG and cytokines. The inhibition of the glutamine transporter SLC1A5 or key enzymes in glutamine (Gln) metabolism results in reduced IgM production in B cells [134].

Research indicates that a high-carbohydrate diet can induce significant alterations in the transcriptional program of γδ T cells within just five days, leading to remodeling of the intestinal epithelium in mice [135]. While αβ T cells comprise over 95% of the T cell population, γδ T cells, though less abundant, are capable of rapidly initiating innate immune responses. Additionally, increased dietary carbohydrate intake elevates the proportion of B cells in the spleen, mesenteric lymph nodes, and Peyer’s patches and enhances the production of antigen-specific IgG post-immunization. Compared to fructose, glucose serves as a superior substrate for B cell development by reducing apoptosis through the mTOR signaling pathway. Consequently, in autoimmune diseases such as rheumatoid arthritis, reducing carbohydrate intake may help mitigate excessive immune responses [136].

Research on the impact of dietary fats on immune system function has predominantly focused on specific types of fatty acids (FAs), which play crucial roles in the body as energy sources, components of biological membranes, and energy storage. As key metabolic products, FAs are integral to immune activation. Among known fatty acid transport proteins, CD36 is one of the most extensively studied. In mice, CD36 accounts for approximately 50% of fatty acid uptake in adipose and muscle tissues. Compared to wild-type mice, CD36-deficient mice exhibit reduced levels of specific IgM and IgG. He [137] et al. demonstrated that CD36-deficient B cells have significantly reduced plasma cell formation, proliferation, mitochondrial mobilization, and oxidative phosphorylation, resulting in weakened germinal center responses, impaired class switching, and decreased antibody production. In activated B cells of mice, the de novo fatty acid synthesis pathway is significantly upregulated, with marked increases in the expression of key genes involved in fatty acid biosynthesis [138]. Additionally, studies have shown that short-chain fatty acids (SCFAs) derived from the gut microbiota can modulate B cell class switching and autoantibody production, thereby ameliorating disease activity and extending survival in lupus mice [139]. Butyrate, a specific type of SCFA, has been shown by Hao et al. to be converted into butyryl-CoA (BCoA), which enhances the activity of CPT1A by antagonizing the binding of the metabolic intermediate malonyl-CoA (MCoA), thereby promoting fatty acid oxidation (FAO) and the differentiation of inducible regulatory T cells (iTregs) [140]. Long-chain fatty acids (LCFAs) require activation by acyl-CoA synthetase long-chain family members (ACSLs) for further metabolism and physiological function. ACSL5 enhances CD8^+^ T cell-mediated cytotoxicity by modulating major histocompatibility complex class I (MHC-I) molecule-mediated antigen presentation in tumor cells [141]. Linoleic acid, a long-chain fatty acid, enhances the antitumor effects of T cells by promoting their differentiation into memory T cells rather than activating CD8^+^ T cells through typical markers, such as GZMβ, IFN-γ, TNF-α, IL-2, and perforin [142]. Myristic acid, a long-chain saturated fatty acid, is significantly downregulated during viral infections and specifically inhibits STING-mediated type I interferon production while enhancing STING-dependent autophagy [143]. Intriguingly, intracellular lipid droplets, essentially reservoirs of ‘oil’, possess a variety of functions. As a major nutrient source, they not only support mitochondrial β-oxidation, providing energy for the body, but also recruit a range of innate immune proteins, contributing to antibacterial defense [144]. In summary, altering dietary fat content and the ratio of saturated to unsaturated fatty acids can significantly impact the lipid composition of lymphocyte membranes, thereby affecting lymphocyte function. The levels and proportions of polyunsaturated fatty acids, particularly essential fatty acids, play a crucial role in modulating both cellular and humoral immune responses [145].

##### Micronutrients

Vitamins, as essential dietary nutrients, also function as key modulators of immune responses. Vitamin deficiencies can lead to elevated inflammation and weakened immunity, while appropriate supplementation may reduce the impact of pathogenic infections. Vitamin A not only possesses anti-inflammatory properties but also regulates thymus and bone marrow homeostasis, thereby enhancing immune function and fortifying the body’s defense against infections [146]. The relationship between Vitamin C and immunity has been a significant area of study. As a potent antioxidant, Vitamin C promotes the proliferation of T cells and NK cells, thereby influencing their immune functions. It has been proposed as a nutritional supplement for various diseases, including cancer [147]. Vitamin D not only promotes the differentiation of monocytes into macrophages but also enhances macrophage chemotaxis and phagocytic function [148], which helps counteract the immune decline associated with aging. In addition to regulating innate immunity, vitamin D modulates adaptive immunity by influencing cytokine and inflammatory factor release from DCs and T cells. Studies have shown a significant inverse relationship between baseline serum vitamin D levels and the development of RA, with early-stage RA patients exhibiting a higher prevalence of severe vitamin D deficiency compared to healthy individuals [149]. Vitamin E, a widely used fat-soluble antioxidant, enhances DC activation and function, promoting the infiltration and activation of tumor antigen-specific T cells. This suggests that vitamin E augments antitumor immune responses by enhancing DC activity [150].

Mineral deficiencies can lead to various diseases, affecting appetite and subsequently digestion, absorption, metabolism, and growth, which indirectly impacts the immune system [151]. For example, calcium deficiency leads to reduced immune cell activity and antibody secretion, making children more susceptible to recurrent infections and the elderly more prone to illness due to weakened immunity. Zinc is crucial for maintaining skin health, controlling the secretion and production of immune regulatory factors, influencing lymphoid organ development, and synthesizing antibodies. Additionally, zinc is involved in nucleic acid and protein metabolism, indirectly affecting immune function [152].

##### Probiotics and Prebiotics

The gut microbiota continuously transforms dietary components into numerous bioactive metabolites, thereby mediating the regulatory effects of diet on host health. Through complex interactions with immune cells in the small intestine and colon, the gut microbiota modulates T and B cells, influencing immune responses in both the peripheral and central nervous systems. For example, the gut microbiota can induce the transcription factor HNF4γ in small intestinal intraepithelial lymphocytes (IELs) of mice via linoleic acid (LA), regulating IL-18 signaling within cells and promoting the development of CD4^+^CD8αα^+^ IELs, thus impacting mucosal immunity in the mouse gut [153]. Probiotics and prebiotics are strategies targeting the microbiota to improve host health. In recent years, a variety of therapeutic approaches have emerged in which probiotics also play a large role in anti-inflammatory regulation and immune cell modulation.

Fecal microbiota transplantation from 8-week-old mice into naturally aging mice over 24 months old has been shown to rejuvenate the gut microbiota and significantly alleviate age-related physiological changes [154]. Additionally, supplementation with *Akkermansia*, *Bifidobacterium*, *Lactobacillus*, or acetate can beneficially modulate the gut microbiota, extending the lifespan of Caenorhabditis elegans or mice and improving age-associated diseases [155]. Various bacterial strains, including specific *Lactobacillus* and *Bifidobacterium bifidum*, have been demonstrated to influence the intestinal mucosal barrier, luminal environment, and mucosal immune system. These probiotics can modulate diverse cells within both the innate and acquired immune systems, such as monocytes, macrophages, dendritic cells, NK cells, and lymphocytes [7]. The anti-inflammatory cytokines they release in the gastrointestinal tract play a crucial role in this immune modulation. Among immunological agents, T regulatory cells (Tregs) are pivotal in maintaining immune regulation and tolerance. Strains like *L. reuteri* and *L. casei* enhance anti-inflammatory responses by elevating IL-10 levels and activating Tregs [156]. Furthermore, innovative probiotics, such as *L. rhamnosus* GG and *L. reuteri* DSM 17938, have been shown to inhibit the activation of T cells and NK cells, as well as the release of IFNγ in cultures of PBMCs exposed to Staphylococcus aureus. This indicates that specific probiotic strains can either suppress or stimulate NK cell activity, underscoring their potential in targeted immune therapies [157]. Therefore, various probiotics and prebiotics can improve immune processes by modulating the gut microbiota, thereby reducing the likelihood and severity of age-related diseases.

##### Caloric Restriction

Caloric restriction has been identified as a pivotal intervention for ameliorating age-related inflammatory changes. Detailed single-cell transcriptomic analyses from studies on aging rats have shown that reducing caloric intake can diminish age-associated inflammation. This reduction in calories notably reverses the pro-inflammatory polarization of M1-type macrophages, increases the proportion of anti-inflammatory macrophages with high phagocytic capabilities, and modifies ligand-receptor interactions between macrophages and stromal cells [59]. In a significant population-based trial in the United States, adults maintaining a 25% calorie-restricted diet over two years experienced a 2% to 3% reduction in biological aging markers, which translates into a 10% to 15% lower mortality risk [158]. Despite challenges in maintaining high levels of calorie restriction due to increased cortisol production and perceived stress, subsequent studies have demonstrated that a 14% calorie reduction does not compromise immune function but rather enhances the immune profile by slowing immune aging and promoting the production of thymic T cells, and PLA2G7 has been identified as a potential anti-aging target [159].

#### 5.1.2. Exercise

The acceleration of aging due to sedentary lifestyles can be significantly countered by replacing inactive periods with 30 min of daily low- or moderate-intensity exercise. This practice is particularly beneficial for individuals over 60, where changes in physical activity levels have a pronounced effect on phenotypic aging [160]. Research indicates that physical activity mitigates senescence and inflammatory markers in both murine models and humans, underscoring exercise’s role in countering the adverse effects of poor dietary habits and sedentary behaviors [161,162,163]. Additionally, these data suggest that exercise can offset the deleterious aspects of senescence brought about by poor diet and sedentary lifestyles.

#### 5.1.3. Sleep

Aging is often accompanied by a decline in sleep quality, which is especially noticeable in middle-aged and older adults. However, the causal relationship between sleep and accelerated aging remains controversial. Elucidating the causal relationship between aging and sleep is greatly limited by the lack of precise measurements of aging. Studies suggest that poor sleep quality may accelerate biological aging, while improved sleep can mitigate the aging acceleration, particularly those accelerations exacerbated by environmental factors like air pollution [164]. Additionally, maintaining a regular sleep routine of approximately 7 h per night can significantly promote both physical and mental health in the aging population [165]. Furthermore, research in older mice highlights the phenomenon of sleep fragmentation, characterized by frequent sleep–wake transitions. Specifically, this condition is linked to a reduction in Hcrt neurons and an increase in their compensatory discharge frequencies, which enhance arousal. In Alzheimer’s disease patients, the loss of Hcrt neurons is more pronounced, leading to increased neuronal excitability during disease progression, compromising sleep quality and possibly contributing to the accumulation of β-amyloid, thus exacerbating the aging process [166].

### 5.2. Pharmacological Interventions

In addition to changing lifestyle, medications are a more precise and expedient approach to addressing the effects of aging. 

Anti-inflammatory treatments, targeting molecules like IL-1β, TNF-α, IFNAR1, caspase-1, and NLRP3, have demonstrated effectiveness in reducing both normal and accelerated aging in murine models [167,168,169,170]. Research indicates that the aging immune system is not inherently weaker; rather, it is restrained by a specific subset of follicular helper T cells (Tfh) that produce interleukin 10 (IL-10). Blockading IL-10 at the time of vaccination in older mice has been shown to restore their antibody responses almost to the levels observed in their younger counterparts [171]. At the cellular level, high-throughput compound screening has identified a TERT activator compound (TAC). This molecule, by activating telomerase reverse transcriptase, promotes TERT transcription in adult somatic cells of humans and mice. It restores youthful expression levels, supports telomere synthesis, and reduces DNA damage signaling at telomeres, effectively reversing signs of cellular aging [172]. Additionally, TPCA-1, a dual inhibitor of NF-κB and Jak-Stat pathways, significantly enhances the expression of the transcription factor Bcl11b, which diminishes from young to early aging stages. This restoration confers youthful characteristics to mammary cells and substantially lowers the incidence of breast cancer, which typically increases with age [173]. Additionally, based on certain clinical outcomes, rapamycin and metformin appear to hold the most promise for practical application. Rapamycin and mTOR inhibitors can regulate cell growth and various cellular processes by reducing the activity of mechanistic target of rapamycin complex 1 (mTORC1). Metformin, an antidiabetic drug, interacts with several known longevity pathways, with effects similar to those of caloric restriction, including enhanced insulin sensitivity [174]. It has also been shown to induce autophagy in CD4^+^ T cells, shifting them from an inflammatory to a non-inflammatory state, and inhibiting Th17 cell differentiation, thus presenting an approach to the mitigation of aging effects [175]. Currently, however, the clinical application of these drugs is often limited by their toxic side effects, especially in elderly patients with comorbidities, as they may increase the risk of hyperglycemia, hyperlipidemia, nephrotoxicity, impaired wound healing, thrombocytopenia, and immunosuppression. Additionally, these small molecule drugs pose a metabolic burden and challenge to liver function in the elderly [174].

### 5.3. Immunomodulatory Therapies

Healthy lifestyles, such as regular exercise and calorie-restricted diets, have been shown to effectively combat aging and age-related diseases. In recent years, both small molecule pharmacologic interventions and genetic approaches have emerged as promising strategies for age protection. However, these interventions all have limitations. Small molecules often lack specificity, potentially affecting non-target cells and tissues, and may inadvertently inhibit anti-apoptotic pathways, risking off-target effects on healthy tissues. Genetic interventions, while potentially effective, carry the risk of long-term adverse effects, such as tumorigenesis. Immunotherapeutic strategies for cell delivery systems are currently of great interest in the treatment of malignant tumors [176], e.g., the precise delivery of mRNA can target cancer [177]. Thus, there is an urgent need to develop accurate immunotherapy interventions for aging.

Precision immunotherapy presents a promising approach to combating aging and related diseases. Maintaining the number and activity of immune cells is crucial for anti-aging. For example, transferring splenocytes from young mice to ERCC1 knockout mice, which have impaired DNA repair, has been shown to slow aging in the latter. This indicates that senescent immune cells may promote systemic aging, while young immune cell transplantation can decelerate it [178]. Vaccination targeting specific antigens represents a promising strategy to combat aging and age-related diseases by inducing tailored immune responses. For instance, Alzheimer’s vaccines reduce Aβ and tau proteins in the brain via antibody production. Targeting molecules like DPP4, IL1-β, and ApoB can slow age-related vascular changes. A CD153 vaccine targets T cells, selectively eliminating pathogenic, highly active, or senescent ones. This targeted clearance of senescent cells may yield more effective outcomes than addressing systemic dysfunction [179]. Immunotherapies for T cell senescence, like CAR-T therapy, which was originally developed for cancer, are gaining traction. CAR-T cells can be engineered to specifically target and eliminate senescent cells, potentially reducing “inflammaging” and enhancing tissue function. Examples include uPAR-targeted CAR-T, which selectively removes senescent cells, rejuvenating aged mice and slowing aging in younger mice [180], and NKG2D-CAR-T, which is capable of targeting NKG2DL-expressing senescent cells in both humans and mice [181]. Due to T cell memory, a single CAR-T administration could provide long-term treatment and preventive benefits; further research is needed to verify its effectiveness in the elderly. Additionally, mTOR inhibitors targeting downstream pathways of the rapamycin complex show potential for the delaying of age-related diseases by modulating T cell function. They enhance immune responses and reduce infection rates in the elderly by decreasing PD1^+^ T cell exhaustion and boosting IFN-stimulated gene expression. These inhibitors are generally well tolerated [87]. With the increase in age, the hematopoietic system as the initial source of immune cells will also be affected by aging, which will accelerate the aging of the body [182]. To counteract age-related diseases, manipulating hematopoietic stem cells (HSCs) has been proposed as a potential strategy [183]. One promising approach involves reprogramming HSCs into induced pluripotent stem cells and then re-differentiating them into youthful HSCs [184].

**Figure 5:**
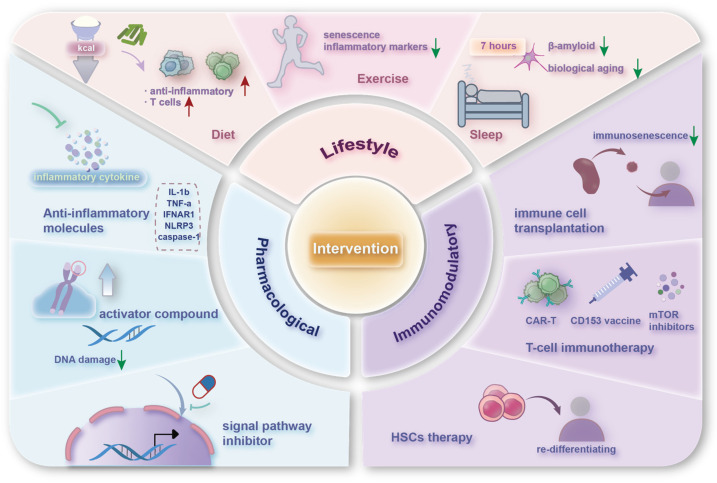
Various potential interventions and strategies that can improve aging immune functions. (1) Lifestyle interventions include adjustments in diet, exercise, and sleep patterns. (2) Pharmacological interventions encompass anti-inflammatory drugs, activators of specific molecules, and inhibitors of certain pro-aging signaling pathways. (3) Immunotherapeutic approaches involve the transplantation of immune cells, CAR-T technologies, and hematopoietic stem cell transplants. The red upward arrow indicates an increase, the green downward arrow indicates a decrease.

Aging is a modifiable process, potentially influenced by nutritional and pharmacological interventions, and immune aging shares this plasticity. As a major contributor to the onset of chronic diseases, interventions targeting either age-related diseases or the aging process itself hold significant promise for promoting healthy aging (Figure 5). Among these strategies, lifestyle changes, particularly dietary adjustments, are the safest and most controllable and are suitable for the everyday modulation of immune function. Pharmacological and immunomodulatory strategies, while more precise and effective, might carry risks of unknown side effects. Therefore, they are generally considered for urgent situations, such as in the management of age-related diseases, where targeted interventions can address specific dysfunctions. 

## 6. Discussion and Perspectives

The immune system is influenced by both intrinsic and extrinsic factors and undergoes significant alterations throughout the aging process, leading to immunosenescence. This term refers to the pathological mechanisms underlying various diseases, including infections, autoimmune disorders, and cancers. Immunosenescence is affected by factors such as thymic involution, inflammation, and DNA damage, which activate inflammation-related signaling pathways, ultimately contributing to disease onset and further influencing individual aging. Notably, the aging of the immune system is plastic [185]; both non-genetic environmental factors and pharmacological interventions can modify or accelerate its progression. Exploring these dimensions enhances our understanding of immunosenescence and establishes a foundation for the application of aging intervention strategies.

Although we have discussed the mutual influence between physiological aging and immunosenescence, their relationship remains elusive. From an immunological perspective, a higher physiological age does not necessarily equate to more severe immunosenescence. The degree of immune aging is more directly linked to the incidence and progression of diseases. Elderly individuals with longevity possess powerful immune systems that can rapidly respond to infections and tumors, thus better defending against infections and slowing the aging process [186]. Therefore, it is indispensable to distinguish between physiological aging and immunosenescence. However, due to the immune system’s complexity, with hundreds of cell types and thousands of epigenetic markers, establishing biomarkers that accurately reflect a patient’s immune status is still an unresolved task [187]. Future research should not only provide a multidimensional assessment of immune capacity, incorporating factors such as cell types, numbers, and functions in both innate and adaptive immunity to inform population immune age, but also conduct longitudinal studies to track changes in immune status over time and validate immune age assessment. The application of multi-omics technologies, which integrate data from genomics, transcriptomics, proteomics, metabolomics, and epigenomics, offers a comprehensive approach to understanding aging at multiple biological levels. These techniques help identify key biomarkers and pathways associated with the aging process, as well as age-related diseases [3,188]. By combining these insights, researchers can uncover how various factors interact to drive aging, enabling more precise interventions, such as personalized medicine, dietary changes, or pharmaceutical treatments, aimed at predicting disease and mortality risk, mitigating the effects of aging, and promoting healthy longevity. 

Despite some advances in medical treatment, one major challenge is the considerable differences in immune responses among species. Many current mouse models are adopted in immunological research, but it remains uncertain whether the interventions that are effective in these models can be applied to humans, as findings from mice do not always predict human outcomes. Many important discoveries have yet to be translated to humans; this is likely due to significant biological differences between species. Moreover, due to unknown factors within the body, even the interventions deemed effective still require long-term studies for validation. Considering the risks and effects of interventions, aside from some safe food-derived substances, other intervention strategies require more fundamental and in-depth clinical research. Additionally, individual variability—such as race, genetic background, and life-style—cannot be underestimated, as it complicates the precise personalization of interventions to slow aging. In summary, the conducting of population studies faces challenges such as ethical concerns, safety issues, and questions of efficacy, all of which require urgent resolution. 

Furthermore, current pharmacological strategies, including small molecule treatments and broader immunotherapies, lack precision in targeting the mechanisms of aging. These approaches generally produce broad effects rather than addressing specific cellular or molecular targets. A major challenge in this field is achieving selective modulation—either activation or inhibition—of particular proteins or cellular pathways without unavoidably disrupting other physiological processes, such as oncogenic pathways. Therefore, developing targeted therapies that may intervene in the aging process without triggering adverse effects remains a complex and critical area of research, while using food-derived substances for precise nutritional interventions is safe, efficient, and highly acceptable.

Ethical considerations surrounding immune cell transplantation or gene interventions in elderly populations must balance the potential benefits and risks. The benefits may include enhanced immune function and the prevention of age-related diseases. However, concerns arise regarding patient consent, autonomy, safety, and long-term effects, especially considering the potential for unforeseen immune responses or complications in the elderly. Additionally, access to these treatments could exacerbate health disparities. A balanced perspective should emphasize both medical advancements and the necessity for rigorous ethical oversight, patient welfare, and equitable access to these interventions.

In summary, this review delves into the multifaceted transformations within the immune system as it ages and examines the quantitative and qualitative shifts in both innate and adaptive immune cells. It highlights how these changes during aging contribute to an increased vulnerability to infections and other health complications. Lastly, it outlines various potential interventions, primarily focusing on nutritional strategies, to mitigate immunosenescence. This review not only enhances our understanding of aging-related immune alterations but also provides perspectives for targeted strategies to enhance immune resilience in the elderly.
